# Ebselen Preserves Tissue-Engineered Cell Sheets and their Stem Cells in Hypothermic Conditions

**DOI:** 10.1038/srep38987

**Published:** 2016-12-14

**Authors:** Ryosuke Katori, Ryuhei Hayashi, Yuki Kobayashi, Eiji Kobayashi, Kohji Nishida

**Affiliations:** 1 Department of Ophthalmology, Osaka University Graduate School of Medicine, 2-2 Yamadaoka, Suita, Osaka 565-0871, Japan; 2 Department of Stem Cell and Applied Medicine, Osaka University Graduate School of Medicine, 2-2 Yamadaoka, Suita, Osaka 565-0871, Japan; 3 Department of Organ Fabrication, Keio University, School of Medicine, 35 Shinanomachi, Shinjuku-ku, Tokyo 160-8582, Japan

## Abstract

Clinical trials have been performed using autologous tissue-engineered epithelial cell sheets for corneal regenerative medicine. To improve stem cell-based therapy for convenient clinical practice, new techniques are required for preserving reconstructed tissues and their stem/progenitor cells until they are ready for use. In the present study, we screened potential preservative agents and developed a novel medium for preserving the cell sheets and their stem/progenitor cells; the effects were evaluated with a luciferase-based viability assay. Nrf2 activators, specifically ebselen, could maintain high ATP levels during preservation. Ebselen also showed a strong influence on maintenance of the viability, morphology, and stem cell function of the cell sheets preserved under hypothermia by protecting them from reactive oxygen species-induced damage. Furthermore, ebselen drastically improved the preservation performance of human cornea tissues and their stem cells. Therefore, ebselen shows good potential as a useful preservation agent in regenerative medicine as well as in cornea transplantation.

Recently, clinical trials have been conducted to evaluate regenerative medicine techniques for treating damaged tissues and organs that have lost their physiological function due to diseases or injuries. In particular, application of the cell sheet technique^1^ has shown successful clinical results for treating serious diseases such as heart failure^2^, esophageal cancer^3^, and corneal stem cell deficiency^4^, and thus, shows good potential as a promising medical treatment.

The cornea consists of three layers, the endothelium, stroma, and epithelium, and the corneal epithelium covers the entire cornea, which functions in maintaining transparency and providing a barrier. MUC16^5^ and ZO-1^6^ are tight junction-related proteins that are essential for maintaining the barrier function of the corneal epithelium. Renewal of the corneal epithelium is carried out by a supply of corneal epithelial stem/progenitor cells located in the corneal limbus^7^; accordingly, reduction in the transparency of the cornea is caused by corneal limbal stem/progenitor cell deficiency (LSCD). Therefore, stem cell transplantation has been performed for treatment of LSCD using a tissue-engineered epithelial cell sheet prepared from culturing autologous oral mucosal stem/progenitor cells^8^. This stem cell-based therapeutic strategy can facilitate the supply of the patient’s own stem/progenitor cells to the damaged tissue that has completely lost its original tissue -stem cells, resulting in much better clinical performance^9^ compared to the conventional treatment of corneal transplantation. Moreover, we have recently reported a novel method for developing human iPS cell-derived corneal epithelial cell sheets, which are therefore expected to be utilized in regenerative medicine^10^.

However, development of a preservation technique for the cell sheets is an essential component to translate this cell sheet transplantation method for standardized and routine clinical practice. Establishing an optimal technique to maintain the cell sheets in good condition can improve the success rate of the transplantation; moreover, it would make it possible to treat patients in a remote area after long-distance transport of cell sheets.

Research on an optimal preservation medium to maintain the viability of tissues and organs has been performed in the field of organ transplantation. For example, University of Wisconsin (UW) solution is commonly used to preserve the liver and kidney^11^, Euro-Collins^12^ and ET-Kyoto solutions^13^ are used to preserve the lungs, and Optisol GS™ is commonly used as a corneal preservation medium. We previously developed a novel screening system to test the effects of candidate preservation media for organs, using luciferase transgenic (*luc tg*) rats that ubiquitously express luciferase^14^. When luciferin, the substrate of luciferase, is injected into *luc tg* rats, excited oxyluciferin is generated to produce luminescence. The resulting emission from this chemical reaction is correlated to the amount of ATP under a condition of sufficient magnesium and luciferin. ATP is the energy currency of cells, and is thus essential for cellular activity; therefore, reduction of ATP leads to cell death. Accordingly, cell viability can be evaluated in a reproducible and sensitive manner by measuring the amount of ATP^15^. Moreover, measuring the amount of ATP in organs and tissues derived from *luc tg* rats is a non-invasive and simple method to evaluate many preservation media simultaneously, because it is possible to measure the ATP levels repeatedly without lysing the cells. Therefore, this system shows good performance for screening the effect of different factors in a preservation medium by measuring their results on the luciferase activity as an index of the remaining amount of ATP. To date, this method has been used to screen several types of preservation media in various organs such as the heart^16^, liver^17^, kidney^18^, islets^19^,^20^, and small intestine^21^. However, to our knowledge, a preservation medium that is ideal for tissue-engineered cell sheets has not yet been screened.

Reactive oxygen species (ROS) accumulate during hypothermic preservation, and are the principal cause of decreasing cell viability and cell membrane desruption^22^. Even in hypothermia, ROS gradually accumulate, though the activity of cell metabolism is reduced. The accumulated ROS provoke DNA damage and cell membrane disruption and finally result in cell sheet destruction.

Thus, in the present study, we used *luc tg* rats to screen potential preservative agents including colloid, sugar, and ROS scavengers which protect the cell membrane, provide the energy required, and remove the ROS, respectively^13^,^23^,^24^,^25^,^26^. We further developed a new and effective preservation medium for cell sheets derived from the oral mucosal epithelium and their stem cells, as well as from human corneal tissue.

## Results

### Screening of the basal preservation medium

**Figure 1:**
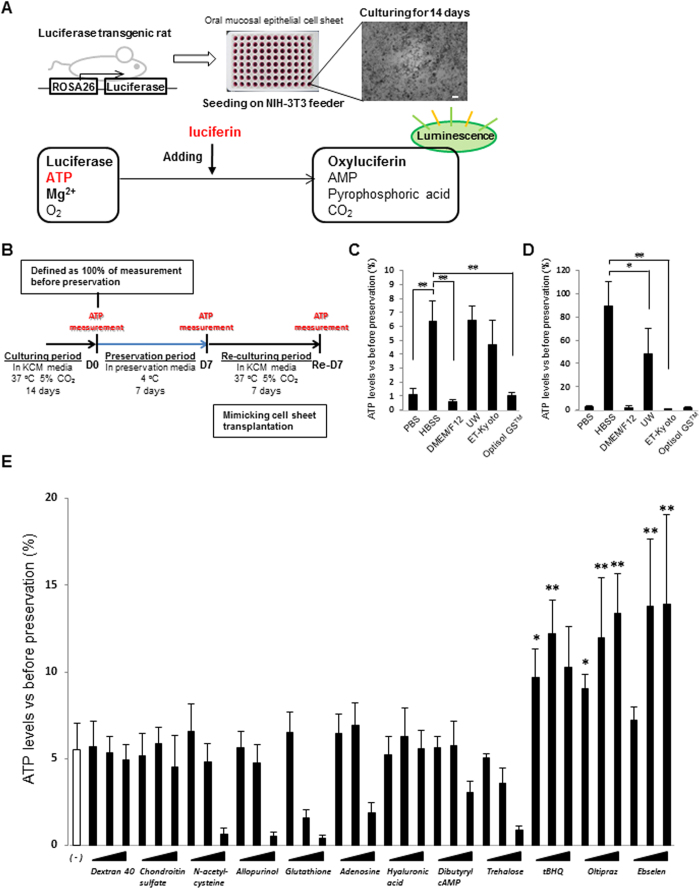
Screening system for determining the optimal preservation medium using oral mucosal epithelial cell (OEC) sheets derived from luciferase transgenic (*luc tg*) rats. (**A**) Luminescence was induced in oral mucosal epithelial cell sheets derived from *luc tg* rats by adding luciferin. The observed luminescence correlates with the ATP levels. Scale bars represent 100 μm. (**B**) Time scale for ATP measurements using OEC sheets derived from *luc tg* rats. ATP levels of the cell sheet were measured at day 0 (D0, before preservation), day 7 ((D7) after preservation), and at 7 days after re-culturing (Re-D7). (**C,D**) Screening of basal medium using OEC sheets derived from *luc tg* rats. ATP levels at (**C**) day 7 after preservation and (**D**) re-culturing were calculated as the ration (%) of the ATP levels measured before preservation. *P < 0.05, **P < 0.001 (versus HBSS). Data are presented as mean ± SD of three independent experiments. (**E**) Screening of HBSS + additives using OEC sheets derived from *luc tg* rats. ATP levels at day 7 after preservation were calculated as the ration (%) of the ATP levels measured before preservation. *P < 0.05, **P < 0.001 (versus HBSS). Data are presented as the mean ± SD of three independent experiments.

In order to determine the basal preservation medium for cell sheets, we screened for phosphate-buffered saline (PBS), Hanks’ balanced salt solution (HBSS), Dulbecco’s modified Eagle’s medium (DMEM/F12), UW solution, ET-Kyoto solution, and Optisol GS™ using oral mucosal epithelial cell (OEC) sheets derived from *luc tg* rats (Fig. 1A). After 7 days of preservation in the respective medium at 4 °C, the ATP levels were measured in the OEC sheets. As shown in Fig. 1C, HBSS, UW solution, and ET-Kyoto solution showed higher ATP levels at day 7 of preservation than those shown by the other preservation media. In contrast, significant decrease in ATP levels was observed in PBS, DMEM/F12, or Optisol GS^TM^. Furthermore, after an additional 7 days of re-culturing, the ATP levels were substantially highest in the OEC sheets preserved in HBSS, even showing a statistically significant difference from the levels in UW solution or ET-Kyoto-preserved cell sheets (Fig. 1D). These results suggested that HBSS can maintain viability of cell sheets. Thus, HBSS is the most suitable basal preservation medium for cell sheets.

### Screening of potential additives for the preservation medium

In order to identify additives for enhancing the preservative effect of HBSS, several additives, including colloids, sugars, reactive oxygen species (ROS) scavengers, energy sources, and Nrf2 activators, were screened to find the ideal candidate for supplementation of HBSS as the basal medium for a new preservation medium for cell sheets. In comparison to the basal medium (HBSS) alone, after 7 days of preservation, significant decreases in the ATP levels were observed with 10 mM N-acetylcysteine (P < 0.001), 10 mM allopurinol (P < 0.001), 30 mM glutathione (P < 0.001), 3 mM glutathione (P < 0.05), 5 mM adenosine (P < 0.05), and 1200 mM trehalose (P < 0.05). By contrast, the ATP levels were significantly increased in OEC sheets preserved in the Nrf2-activating agents tert-butylhydroquinone (tBHQ), oltipraz, and ebselen. In particular, ebselen only resulted in significantly increased ATP levels compared to HBSS at concentrations of 10 μM (P < 0.001) and 100 μM (P < 0.001) (Fig. 1E). Overall, these results suggested that the Nrf2-activating agents, tBHQ, oltipraz, and especially ebselen could effectively maintain the viability of the preserved cell sheets for 7 days at 4 °C.

### Harvesting of human OEC (hOEC) sheets from a temperature-responsive culture dish

**Figure 2:**
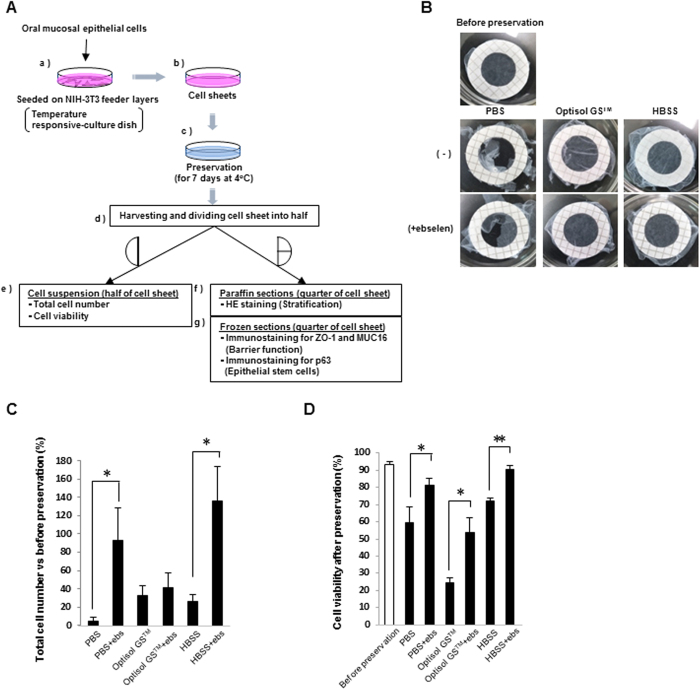
Total cell number and viability of hOEC sheets before and after preservation. (**A**) Overall methods for preservation and preparation of cell sheets for analysis. hOEC sheets were cultured for 14 days and then preserved for 7 days at 4 °C. After preservation, hOEC sheets were harvested and divided for each analysis. (**B**) Harvesting of hOEC sheets before and after preservation. (**C**) Total cell numbers after preservation vs before preservation. The total cell numbers after preservation were calculated as the ratio (%) relative to the number of cells counted before preservation. *P < 0.05 (within a medium with or without ebselen [ebs]). Data represent the mean ± SD of three independent experiments. (**D**) Viability of cell sheets before and after preservation. *P < 0.05, **P < 0.001 (within a medium with or without ebselen). Data represent mean ± SD of three independent experiments.

We next examined whether hOEC sheets could be harvested intact from a temperature-responsive culture dish after preservation, and subsequently investigated as shown in Fig. 2A(e–g). The hOEC sheets preserved in PBS or PBS + ebselen could not be harvested due to irreversible damage. In contrast, hOEC sheets preserved in Optisol GS™, Optisol GS™ + ebselen, HBSS, or HBSS + ebselen were well-preserved, and did not show any sign of damage during harvesting (Fig. 2B).

### Effect of ebselen on cell numbers and viability of hOEC sheets during 7 day preservation

To confirm the beneficial effect of ebselen on cell sheet preservation, hOEC sheets were soaked in HBSS, PBS, and Optisol GS™ with or without ebselen for 7 days, cell number and viability of the cell sheets were compared. The addition of ebselen had a significant effect on increasing the cell number in sheets preserved in PBS or HBSS, although there was no difference for sheets preserved in Optisol GS™ (Fig. 2C). Furthermore, the viability of the hOEC sheets was evaluated after preservation in these media with or without ebselen supplementation. The viability decreased in all cases compared to the levels before preservation; however, for all media, the viability of cell sheets was significantly increased with ebselen supplementation (Fig. 2D). In particular, the viability and the cell number were highest when the sheets were preserved in the HBSS + ebselen medium. Therefore, under preservation conditions of 7 days at 4 °C, HBSS + ebselen was determined to be the best candidate preservation medium for hOEC sheets.

### Morphology of the hOEC sheets after preservation

**Figure 3:**
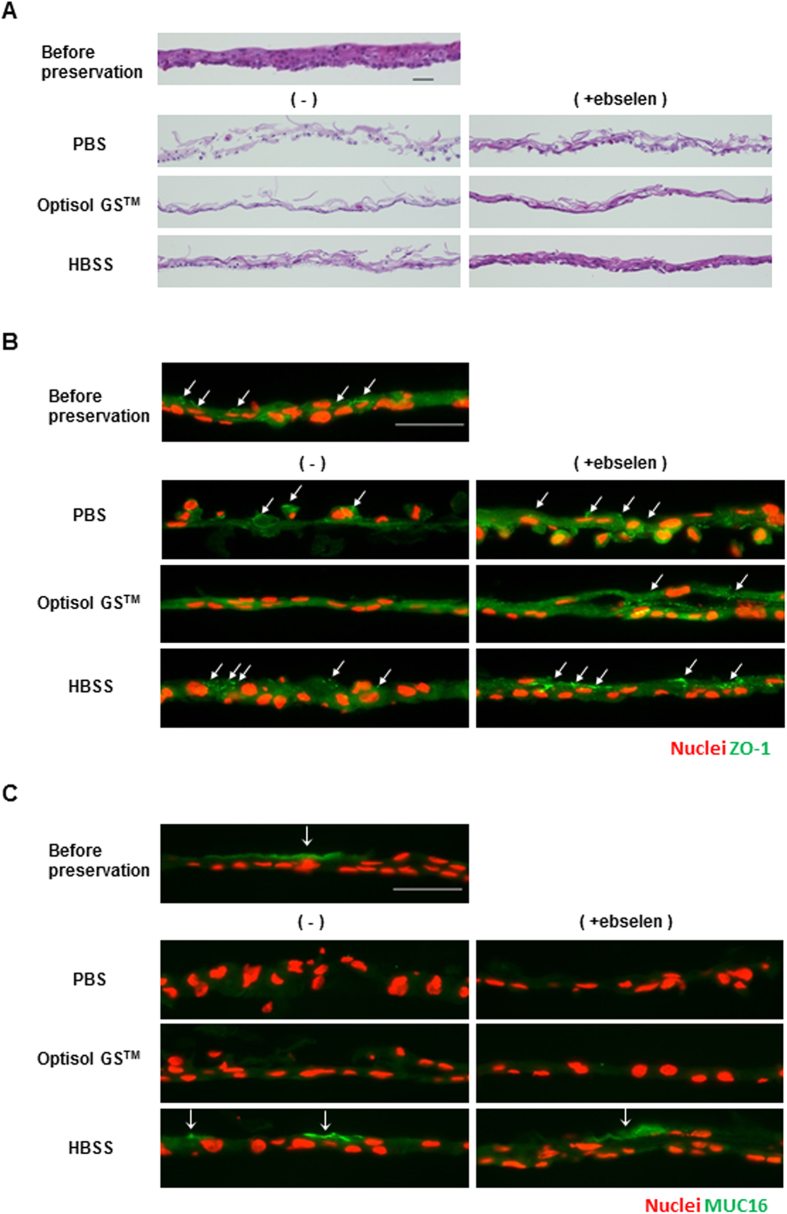
Morphology and expression of ZO-1 and MUC16 in hOEC sheets before and after preservation in PBS, Optisol GS^TM^, or HBSS with or without ebselen. (**A**) Hematoxylin and eosin staining of hOEC sheets before and after preservation. Scale bars represent 50 μm. (**B**) Images of ZO-1 (green) expression and nuclei (red) of hOEC sheets before and after preservation. Scale bars represent 50 μm. (**C**) Images of MUC16 (green) expression and nuclei (red) of an hOEC sheet before and after preservation. Scale bars represent 50 μm.

The morphology of the hOEC sheets before and after preservation was observed using hematoxylin and eosin (HE) staining (Fig. 3A).

In PBS or PBS + ebselen, cell-cell junctions clearly collapsed due to loss of cell sheet formation. In Optisol GS™, the upper cells in the hOEC sheet were lost, and the majority of cell sheet had formed into a monolayer state. However, the addition of ebselen to Optisol GS™ increased, the staining intensity of eosin, which indicated the presence of cytoplasm. In both HBSS and HBSS + ebselen, the morphology and stratification of the cell sheets were well maintained, and the staining intensity of eosin was improved by ebselen supplementation. These results suggested that the cell membrane and extracellular matrix were improved by the addition of ebselen.

### Expression of ZO-1, MUC16, and p63 before and after preservation for 7 days

ZO-1 is the lining protein of the tight junction and is associated with cell-to-cell barrier function. ZO-1 was found to be expressed in the cell sheets preserved in HBSS; however, staining for ZO-1 was weak for hOEC sheets preserved in PBS, and was entirely absent in those preserved in Optisol GS™ (Fig. 3B). Furthermore, addition of ebselen to each medium resulted in strong ZO-1 expression of the hOEC sheet.

MUC16 is a membrane-associated mucin that is expressed on the surface of the corneal stratified epithelium, has water-holding capacity, and is related to maintenance of barrier function. The staining results demonstrated that MUC16 was expressed in the hOEC sheets preserved in HBSS or HBSS + ebselen (Fig. 3C); however, it was not expressed in the hOEC sheets preserved in the other media, owing to loss of the surface epithelium. Thus, no beneficial effect of ebselen on MUC16 expression could be observed in hOEC sheets preserved in PBS or Optisol GS^TM^.

**Figure 4:**
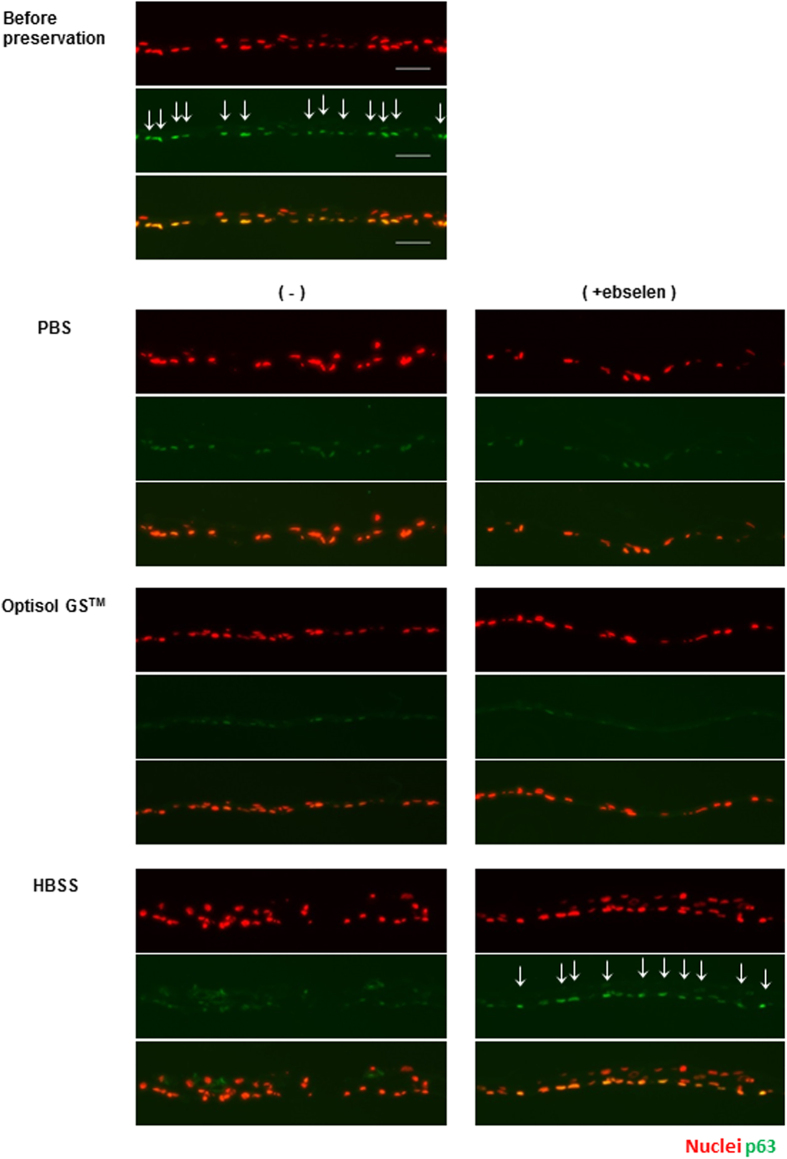
Expression of p63, the epithelial stem cell marker, in hOEC sheets before and after preservation in PBS, Optisol GS^TM^, or HBSS with or without ebselen. Images of p63 (green) expression of and nuclei (red) hOEC sheets before and after preservation. Scale bars represent 50 μm. Images of nuclei (top; red), p63 (middle; green) and superimposing nuclei and p63 (bottom).

The epithelial stem cell marker, p63, was expressed in the basal cells of hOEC sheets before preservation (Fig. 4). The expression of p63 was observed in hOEC sheets before preservation and in those preserved in HBSS + ebselen. In contrast, the expression was faint in hOEC sheets preserved in the other media. These data suggest that HBSS + ebselen maintained the expression of ZO-1 and MUC16, and further prevented the reduction of p63 expression in hypothermic preservation.

### Colony-forming assay for hOEC sheets after 7-day preservation

**Figure 5:**
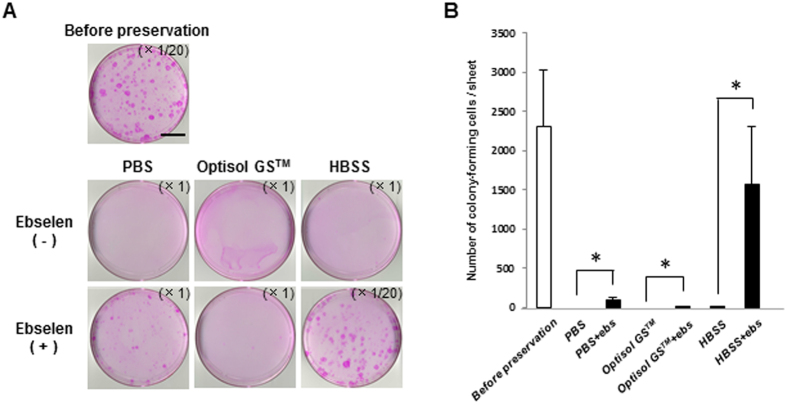
Colony-forming assay of hOEC sheets before and after preservation in PBS, Optisol GS^TM^, or HBSS with or without ebselen. (**A**) Representative images of colonies in an hOEC sheet before and after preservation. Scale bars represent 25 mm. (**B**) Number of colony-forming units in the hOEC sheet before and after preservation. *P < 0.05 (for comparisons within a medium with or without ebselen [ebs]). Data represent mean ± SD of three independent experiments.

We performed a colony-forming assay to examine how the stem cells in the hOEC sheets were maintained after 7 days of preservation (Fig. 5).

Almost no colony formation was observed in the hOEC sheets preserved in PBS, Optisol GS™, or even in HBSS. However, the number of colonies formed increased significantly when ebselen was added to the medium in each case, especially for HBSS + ebselen, which was similar to number of colonies observed before preservation. These results confirmed that ebselen has a strong positive effect on the preservation of stem cells in hOEC sheets.

### The reduced/oxidized glutathione (GSH/GSSG) ratio and lactate dehydrogenase (LDH) release in hOEC sheets preserved for 7 days

**Figure 6:**
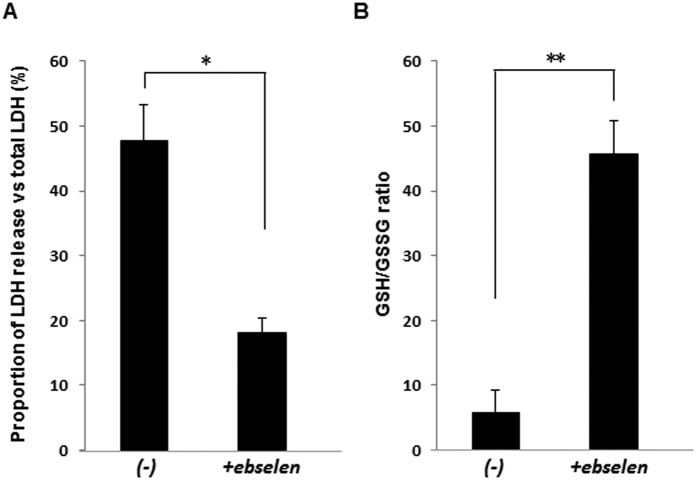
Effect of ebselen on reactive oxygen species generation in hOEC sheets after preservation in HBSS with or without ebselen. (**A**) Proportion of LDH release vs total LDH and (**B**) GSH/GSSG ratio in hOEC sheets after preservation. *P < 0.05, **P < 0.001. Data represent the mean ± SD of three independent experiments.

The results described above clearly demonstrated that ebselen supplementation could improve the ATP levels, cell number, and cell viability under conditions of hypothermic preservation. To clarify the mechanism underlying the effect of ebselen, we examined LDH release and the GSH/GSSG ratio of the hOEC sheets after hypothermic preservation. After 7 days of preservation, the proportion of LDH release had reduced to less than half, and the GSH/GSSG ratio was increased by more than 6-fold in HBSS + ebselen compared to that preserved in HBSS (Fig. 6). These results indicate that ebselen could suppress cell membrane injury by reducing ROS generation during hypothermic preservation.

### Effect of ebselen on the nuclear translocation of Nrf2 in the re-culture period of hOEC sheets preserved in HBSS

Since ebselen is known to be an Nrf2 activator, we investigated whether ebselen activated Nrf2 in the preserved hOEC sheets by examining the nuclear translocation of Nrf2 after preservation. After 7 days of preservation, the medium was changed to keratinocyte culture medium (KCM) for re-culturing, and the nuclear translocation of Nrf2 was monitored at 0, 1, 3, and 6 hours of re-culturing by immunostaining.

**Figure 7:**
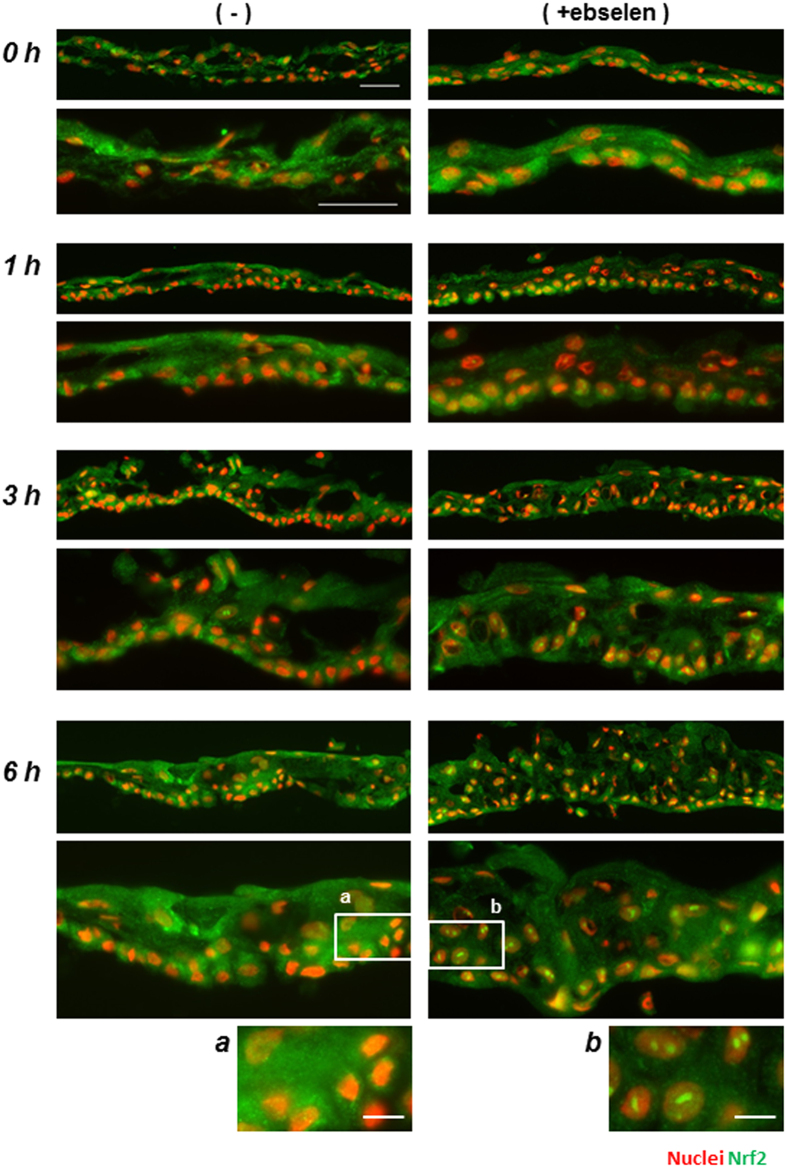
Effect of ebselen on Nrf2 nuclear translocation in hOEC sheets during re-culturing. Images of Nrf2 (green) nuclear translocation of hOEC sheets during re-culturing. The magnification is ×200 (top) and ×400 (bottom) at each time point. Scale bars represent 50 μm. Images (**a**) and (**b**) represent magnified insets at the 6-hour time point (×200). Scale bars represent 10 μm.

Nuclear translocation of Nrf2 was weakly detected after 3 and 6 hours of re-culturing in the hOEC sheets preserved in HBSS, and was intensely detected after 1, 3 and 6 hours of re-culturing in the sheets preserved in HBSS + ebselen. Notably, nuclear translocation was more frequent after 6 hours of re-culturing in the sheets preserved in HBSS + ebselen; however, it was not detected immediately after preservation (at 0 hours of re-culturing) in either medium (Fig. 7).

### Preservation of human corneal limbal tissue in Optisol GS™ and HBSS + ebselen

**Figure 8:**
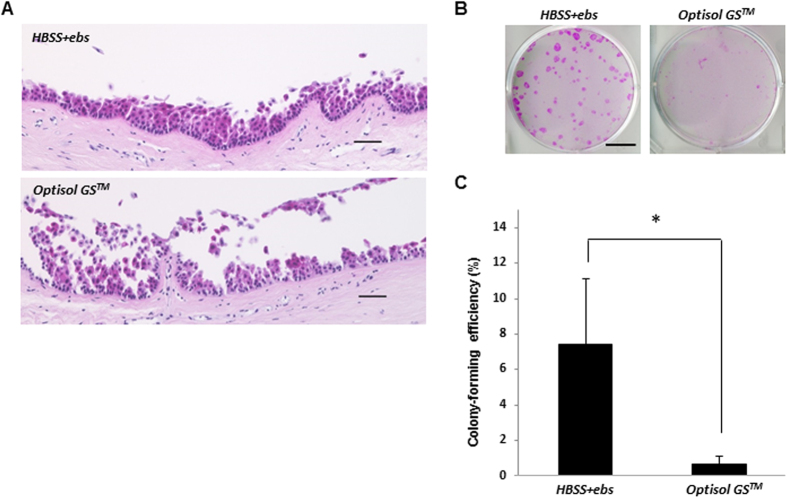
Morphology and colony-forming assay of human corneal limbal tissue after preservation in HBSS + ebselen or in Optisol GS^TM^. (**A**) Hematoxylin and eosin staining of human corneal limbal tissues preserved in Optisol GS™ or HBSS + ebselen. Scale bars represent 50 μm. (**B,C**) Colony-forming assay of human corneal limbal tissues preserved in Optisol GS™ or HBSS + ebselen. Representative images of colonies in corneal limbal tissues after preservation (**B**). Scale bars represent 15 mm. *P < 0.05 was considered significant. Data represent the mean ± SD of four independent experiments (**C**).

The results described thus far show that ebselen improved the preservation performance for OEC sheets containing stem cells. In turn, to examine the effects of ebselen in the preservation of corneal limbal tissues, we assessed the colony-forming ability and morphology of HE-stained corneal limbal tissues after preservation in Optisol GS™ or HBSS + ebselen (Fig. 8). After preservation in Optisol GS™, a large part of the corneal limbal epithelial region was lost, and the stromal tissue was partially exposed. In contrast, after preservation in HBSS + ebselen, the limbal epithelial layer was well-maintained and showed normal morphology (Fig. 8A).

Human corneal limbal tissue preserved in HBSS + ebselen showed significantly higher colony-forming efficiency compared to that preserved in Optisol GS™. This indicates that HBSS + ebselen could effectively maintain corneal limbal stem cells in conditions of hypothermic preservation (Fig. 8B).

## Discussion

In the present study, we screened candidate preservation media for OEC sheets, and successfully demonstrated that the organic selenium compound ebselen, a known Nrf2-activating agent, shows good potential for the preservation of OEC sheets and their stem cells under hypothermia.

First, we determined the optimal basal medium for subsequent screenings for a preservation medium. HBSS showed good effects on maintaining cell numbers and viability. Given its simple formulation of PBS supplemented with magnesium, calcium, and glucose, this result suggests that Mg^2+^ and Ca^2+^, which are involved in cell adhesion, are also key factors for maintaining the epithelial structure and morphology of cell sheets in hypothermic preservation conditions (Supplemental Fig. 1). By contrast, Optisol GS™ and DMEM/F12 did not show good preservation ability, even though both of these media also contain Ca^2+^ and Mg^2+^. It is possible that enrichment of nutrients, including amino acids and growth factors, might be an obstacle for good preservation under hypothermic conditions. Collectively, these results demonstrated that HBSS was the most suitable basal preservation medium for the screening study.

The results of screening OEC sheets derived from *luc tg* rats or humans clearly showed that ebselen has good effects on the preservation of cell sheets. Thereby, we examined the potential mechanism underlying these effects. First, we measured LDH release and the GSH/GSSG ratio in hOEC sheets after 7 days of preservation at 4 °C. We showed that ebselen decreased the amount of LDH released, and also increased the GSH/GSSG ratio in hOEC sheets. In general, ROS can induce cell membrane injury via lipid peroxidation and apoptosis following production of hydroxyl radicals from increases in chelatable iron and hydrogen peroxide (i.e., a Fenton reaction)^27^,^28^,^29^. Given the known function of ebselen in Nrf2 activation, these results indicate that ebselen-induced Nrf2 activation exerts an antioxidant and anti-apoptotic effect to protect the cell from ROS-induced damage.

To examine whether Nrf2 activation by ebselen directly contributes to the survival of the cell sheets in hypothermic preservation, we evaluated this potential effect of ebselen via monitoring of Nrf2 translocation to the nucleus; however, Nrf2 translocation could not be detected during hypothermic preservation, even in HBSS containing ebselen. Some previous reports have indicated the direct effects of ebselen such as in (a) biological defense via its glutathione peroxidase-like activity^30^; (b) anti-apoptosis effects upon inhibition of inositol triphosphate (IP3)-dependent calcium release by the endoplasmic reticulum^31^; (c) inhibition of nitric oxide synthases^32^, H^+^ K^+^-ATPase^33^, and Na^+^ K^+^-ATPase^34^; and (d) induction of the expression of GSTP1 protein^35^. These reports suggest that ebselen plays a direct role in protecting OEC sheets and stem cells by removing ROS, having anti-apoptotic effects and suppressing ATP consumption during hypothermal preservation. Moreover, after 3 and 6 hours of re-culturing at 37 °C after 7 days preservation, ebselen induced Nrf2 nuclear translocation in the hOEC sheets. Thereby, it is expected that addition of ebselen to the preservation medium would protect a graft from ROS-induced apoptosis and cell injury after transplantation. These results suggest that ebselen has two different roles in the preservation and re-culturing periods: one exerting an indirect effect via Nrf2 activation during re-culturing after hypothermic preservation, and the other as a direct effect in the active removal of ROS generated in response to hypothermic conditions.

Optisol GS™ is a common preservative medium used for corneal tissues, and has been shown to be particularly suitable for preservation of the corneal endothelium in hypothermia^36^; however, some reports have suggested that the medium is not suitable for the preservation of corneal epithelium^37^,^38^. The results of our experiment clearly showed that Optisol GS™ is not suitable for preservation of the corneal epithelium or its stem cells.

Ebselen was successfully selected by our screening method, and was found to be an effective compound for the hypothermic preservation of human corneal tissues and hOEC sheets, including their stem cells. The ability to maintain stem cells in tissues is a key factor in regenerative medicine or organ transplantation, as an abundant supply of healthy stem cells must be available for damaged tissues. In order to standardize the use of cell sheets for broad applications in regenerative medicine in the near future, feasible and simple preservation techniques are essential. We believe that ebselen is a key molecule for the development of a new preservation technique for tissue-engineered cell sheets, and will greatly contribute to promoting the generalization of regenerative medicine.

## Methods

### Isolation and cultivation of OECs derived from *luc tg* rats

Oral mucosal tissues derived from *luc tg* rats were provided by the Division of Organ Replacement Research Center for Molecular Medicine of Jichi Medical School. All experiments in this study were performed in accordance with Jichi Medical University and Osaka University Medical School Guidelines for experimental animals and recombinant DNA experiments.

For isolation of OECs, the oral mucosal tissues were washed in DMEM (Gibco, Grand Island, NY, USA) containing 1% antibiotic-antimycotic (Gibco), and then immersed in DMEM containing dispase I (Godo Shusei Co., LTD. 1000 pU/mL) at 4 °C for 15 hours. After dispase I treatment, the oral mucosal tissues were neutralized in a 0.02% ethylenediaminetetraacetic acid (EDTA) solution (Nacalai Tesque, Inc. Kyoto, Japan), and washed in PBS. Subsequently, epithelial layers, including stem/progenitor cells, were separated from the tissue using tweezers; then, the separated epithelial cells were isolated by treatment with a 0.25% trypsin-EDTA solution (Gibco) for 20 minutes at 37 °C, and were resuspended and seeded into 6 well plates on a seeding NIH-3T3 feeder at a density of 3–5 × 10^5^ cells per well in keratinocyte culture medium (KCM). The composition of KCM is as follows: DMEM high-glucose medium (Gibco), Nutrient Mixture F-12 (Gibco), 10% fetal bovine serum (Japan Bio Serum), 400 ng/mL hydrocortisone succinate (Wako), 2 nM 3,3′,5- triiodo-l-thyronine sodium salt (MP Biomedicals), 1 nM cholera toxin (List Biological Laboratory), 2.25 μg/mL bovine transferrin HOLO form (Gibco), 2 mM l-glutamine (Gibco), 0.5% insulin transferrin selenium (Gibco), and 10 ng/mL recombinant human epidermal growth factor (R&D Systems). When the cells reached subconfluence, they were trypsinized and resuspended in KCM. The cells were reseeded on NIH-3T3 feeder layers in 96-well white/clear flat bottom plates (BD Falcon™, Franklin Lanes, NJ, USA), and were cultured in KCM for 14 days at 37 °C and 5% CO_2_. The OECs were used at a passage number of 5 or less for the subsequent experiments.

### Culture of human OEC (hOEC) sheets

hOECs (human oral keratinocytes) were purchased from Sciencell^TM^ Research Laboratories (Carlsbad, CA, USA), and cultured in 75 cm^2^ flasks (Corning Inc. NY, USA) until subconfluence in Cnt.24 (CELLnTec advanced cell systems AG, Switzerland). Then, the cells were trypsinized at 37 °C for 10 minutes, counted, and re-seeded on NIH-3T3 feeder layers in 35-mm temperature-responsive culture dishes (UpCell^®^; CellSeed Inc. Tokyo, Japan) at a density of 0.32 × 10^5^ cells/cm^2^, and were then cultured in KCM for 14 days at 37 °C and 5% CO_2_ (Fig. 2A(a,b)).

### Preparation of the preservation media

PBS, HBSS, DMEM/F12 (Thermo Fisher Scientific, Inc.), UW solution (Viaspan^®^; Bristol-Myers Squibb, Co.), ET-Kyoto solution (Otsuka Pharmaceutical Factory, Inc.), and Optisol GS™ (Bausch & Lomb, Rochester, NY) were prepared as candidates for a basal preservation medium. The following additives were also prepared: dextran 40 (0.2, 2, 20 g/L; Wako), chondroitin sulfate A (0.01, 0.1, 1%; Sigma-Aldrich, St. Louis, MO, USA), N-acetylcysteine (0.1, 1, 10 mM; Wako), allopurinol (0.1, 1, 10 mM; Tokyo Chemical Industry Co., Ltd), glutathione (0.3, 3, 30 mM; Sigma-Aldrich), adenosine (0.05, 0.5, 5 mM; Sigma-Aldrich), hyaluronic acid (0.01, 0.1, 0.5%; Calbiochem), dibutyryl cAMP (0.2, 2, 20 mM; Enzo Life Sciences), trehalose (12, 120, 1200 mM; Hayashibara), tert-butylhydroquinone (tBHQ; 0.1, 1, 10 μM; Sigma-Aldrich), oltipraz (1, 10, 100 μM; Sigma-Aldrich), and ebselen (1, 10, 100 μM; Sigma). Each of these additives was added to HBSS individually.

### ATP measurements in OEC sheets derived from *luc tg* rats

As shown in Fig. 1B, ATP measurements were performed at D0, D7 and Re-D7. At first, cell sheets gained by cultivation of OECs derived from *luc tg* rats were washed 3 times in HBSS, and incubated in HBSS for 1 hour at 4 °C. Then, beetle luciferin (Promega Corporation, USA) was added at a final concentration 0.19 mg/mL and photon intensity in the cell sheet was immediately measured before preservation based on the luciferase/luciferin reaction (to estimate the ATP levels) using a luminescence microplate reader (Centro LB960; Berthold Technologies GmbH & Co. KG. After each measurement, each cell sheet was washed 3 times in HBSS, immersed in the preservation medium, and left for 7 days at 4 °C.

The cell sheets were washed 3 times in HBSS after preservation, and the amount of ATP was measured (for comparison at D0 and D7). The medium was changed to KCM after ATP measurement at 7 days of preservation, and the sheets were re-cultivated for 7 days at 37 °C and 5% CO_2_. After 7 days of re-cultivation, the amount of ATP was measured again. The ATP levels were calculated as the ratio (%) of ATP levels measured before preservation.

### Cell sheet harvesting

After the preservation of hOEC sheets, a PVDF membrane (outer and inner diameters of 26 mm and 16 mm, respectively) was placed on the hOEC sheet, and the cell sheets were detached from the peripheral region with a PVDF membrane using tweezers^8^,^39^. For subsequent analyses, the harvested cell sheets were cut into equal halves or quarters.

### Measurement of cell number and viability in preserved hOEC sheets

The harvested cell sheets were cut into equal halves. One quarter was embedded in Tissue Tek O.C.T compound (Sakura) for preparation of frozen sections, and another quarter was used for paraffin sections. The remaining half was used to analyse the cell number and viability by fluorescent-activation cell sorting analysis (FACSVerse; BD Becton, Dickinson & Co.). In brief, the cell sheet was trypsinized at 37 °C for 10 minutes and then dyed in 7-AAD staining solution (BD Pharmingen™) for measurement of survival rate (Fig. 2A(d–g)).

### HE staining of hOEC sheets and human corneal limbal tissues

HE staining was performed on the preserved cell sheets and human corneal limbal tissues to examine the degree of stratification and morphology of the cell sheets. One quarter of the cell sheet and the corneal limbal tissues were fixed with 10% formaldehyde neutral buffer solution (Nacalai Tesque) at room temperature overnight. After washing with distilled water, the cell sheets and corneal limbal tissues were embedded in paraffin and processed into 3-μm-thick sections. The sections were deparaffinized and hydrated, and were stained with HE. Microphotographs were taken with BIOREVO BZ-9000 (KEYENCE, Co.), and the morphology was examined.

### Immunostaining of hOEC sheets from frozen sections

hOEC sheets embedded in Tissue Tek O.C.T compound were sliced into 10-μm-thick sections. The slides were blocked in tris-buffered saline (TBS; TaKaRa Bio, Inc.) containing 5% donkey serum (Jackson ImmunoResearch Laboratories, Inc.), and 0.3% Triton X-100 (Sigma-Aldrich) for 1 hour at room temperature. Then, the sections were incubated with primary antibodies, including mouse monoclonal ZO-1 antibody (1A12, 1:200; Invitrogen), mouse monoclonal MUC16 antibody (Ov185, 1:200; Abcam), mouse monoclonal p63 (4A4, 1:200; Santa Cruz), negative control mouse IgG1 antibody (1:200; Dako An Agilent Technologies, Co.), or rabbit polyclonal anti Nrf2 antibody (1:200; Abcam) at 4 °C overnight, diluted in TBS containing 1% donkey serum and 0.3% Triton X-100. After washing in TBS 3 times, the sections were incubated with Alexa Fluor 488-conjugated donkey anti-mouse IgG antibody (1:200; Invitrogen) as a secondary antibody and bisbenzimide H33342 trihydrochloride (1:100; Sigma-Aldrich) for a nuclear counterstain, and incubated for 1 hour at room temperature. Then, the sections were washed 3 times in TBS and mounted with PermaFluor™ Aqueous mounting medium (Thermo Fisher Scientific, Inc.). Observation was performed with AxioObserver D1 microscope objectives (Carl Zeiss).

### Colony-forming assay in hOEC sheets before and after preservation

Preserved hOEC sheets were trypsinized with 0.25% trypsin-EDTA and resuspended in KCM. For hOEC sheets before preservation and those preserved in HBSS + ebselen, the cell suspensions were seeded to 5% of a single cell sheet on NIH-3T3 feeder layers in 100-mm cell culture dishes (BD Falcon™). hOEC sheets preserved in other media were seeded over the whole cell suspension on NIH-3T3 feeder layers in 100-mm cell culture dishes. The seeding cells were cultivated in KCM for 10 days at 37 °C and 5% CO_2_. Colonies were fixed with a 10% formaldehyde neutral buffer solution (Nacalai Tesque), and stained with 2% rhodamine. The number of colonies was counted and has been represented by bar graphs.

### Measurement of LDH release and the GSH/GSSG ratio

Cell membrane integrity was determined using CytoTox-ONE^TM^ Homogeneous Membrane Integrity Assay (Promega Corporation, USA). Prior to, and after preservation of the cell sheets for 7 days, the supernatants and the cell sheets were collected, and LDH release was quantified. The amount of LDH release was calculated considering an LDH level of 100% for the total of the cell sheet and supernatants.

After preservation for 7 days, the intracellular GSH/GSSG ratio of the hOEC sheet was measured using GSH/GSSG-Glo™ Assay (Promega Corporation, USA) according to the manufacturer protocol. The amount of fluorescence was measured with Multilabel Reader ARVO™X4 (Perkin Elmer).

### Colony-forming assay in human corneal limbal tissues after preservation

Human corneal limbal tissues were obtained from SightLife (Seattle, WA, USA). They were preserved in Optisol GS™ for 10 days, and then each half of the human corneal limbal tissue was individually preserved in Optisol GS™ or HBSS + ebselen for an additional 10 days.

Human corneal limbal tissue preserved for 10 days at 4 °C was treated with DMEM containing 2.6 U/mL dispase for 1 hour at 37 °C. Subsequently, human corneal epithelial cells containing limbal stem/progenitor cells were collected by scratching of the corneal limbus using tweezers. The collected corneal epithelial cells were trypsinized with 0.25% trypsin-EDTA for 10 minutes at 37 °C, resuspended in 5% KCM and seeded on NIH-3T3 feeder layers in 60 mm culture dish (BD Falcon™). After colony-formation, the number of colonies was counted following fixation with a 10% formaldehyde neutral buffer solution, and subsequent staining with 2% rhodamine.

### Statistical analysis

All data are expressed as means ± SD. Results of screening for the preservation medium using *luc tg* rats were compared by analysis of variance (ANOVA). All statistical analyses were performed with JMP^®^ Pro 11.2.1 software (SAS Institute Inc., Cary, NC, USA). P-values less than 0.05 were considered statistically significant.

## Additional Information

**How to cite this article**: Katori, R. *et al*. Ebselen Preserves Tissue-Engineered Cell Sheets and their Stem Cells in Hypothermic Conditions. *Sci. Rep.* **6**, 38987; doi: 10.1038/srep38987 (2016).

**Publisher's note:** Springer Nature remains neutral with regard to jurisdictional claims in published maps and institutional affiliations.

## Supplementary Material

Supplementary Information
