# Supplementary material for: Ebselen Preserves Tissue-Engineered Cell Sheets and their Stem Cells in Hypothermic Conditions
Source: Sci Rep. 2016 Dec 14;6:38987. doi: 10.1038/srep38987 (PMC5155221; doi:10.1038/srep38987)

Ebselen Preserves Tissue-Engineered Cell Sheets and their Stem Cells in Hypothermic Conditions

Ryosuke Katori[1], Ryuhei Hayashi[1, 2], Yuki Kobayashi[1], Eiji Kobayashi[3] & Kohji Nishida[1]

[1]Department of Ophthalmology, Osaka University Graduate School of Medicine, 2-2 Yamadaoka, Suita, Osaka 565-0871, Japan.

[2]Department of Stem Cell and Applied Medicine, Osaka University Graduate School of Medicine, 2-2 Yamadaoka, Suita, Osaka 565-0871, Japan.

[3]Department of Organ Fabrication, Keio University, School of Medicine, 35 Shinanomachi, Shinjuku-ku, Tokyo 160-8582, Japan.

Correspondence and requests for materials should be addressed to K.N. (knishida@ophthal.med.osaka-u.ac.jp)

Supplementary Information

Supplementary Figure Legends

Supplementary Figure S1. Validation (analysis) of hOEC sheets before and after preservation in HBSS or PBS + Mg^{2+} + Ca^{2+} for 7 days at 4°C. (A) Images after harvesting of hOEC sheets before and after preservation. hOEC sheets preserved in HBSS or PBS + Mg^{2+} + Ca^{2+} could be harvested without cell sheets destruction. (B) Total cell number of hOEC sheets after preservation. The total cell number after preservation was calculated as the ratio (%) relative to the number of cells counted before preservation. (C) Viability of hOEC sheets before preservation (92.9%), preserved in HBSS (82.0%) or PBS +Mg^{2+} + Ca^{2+} (78.6%) for 7 days at 4°C. (D) Hematoxylin and eosin staining and (E) expression of ZO-1, MUC16 and p63 in hOEC sheets before and after preservation. hOEC sheets preserved in HBSS or PBS + Mg^{2+} + Ca^{2+} maintained expression of MUC16 (green) and ZO-1 (green), and the form of cell sheets. The epithelial stem cell marker, p63 (green) was expressed in the basal cells of hOEC sheets before preservation, but was faintly expressed in hOEC sheets preserved in HBSS or PBS + Mg^{2+} + Ca^{2+}. Scale bars represent 50μm.

Supplementary Figure S1. Validation of hOEC sheets before and after preservation in HBSS or PBS + Mg^{2+} + Ca^{2+} for 7 days at 4°C.

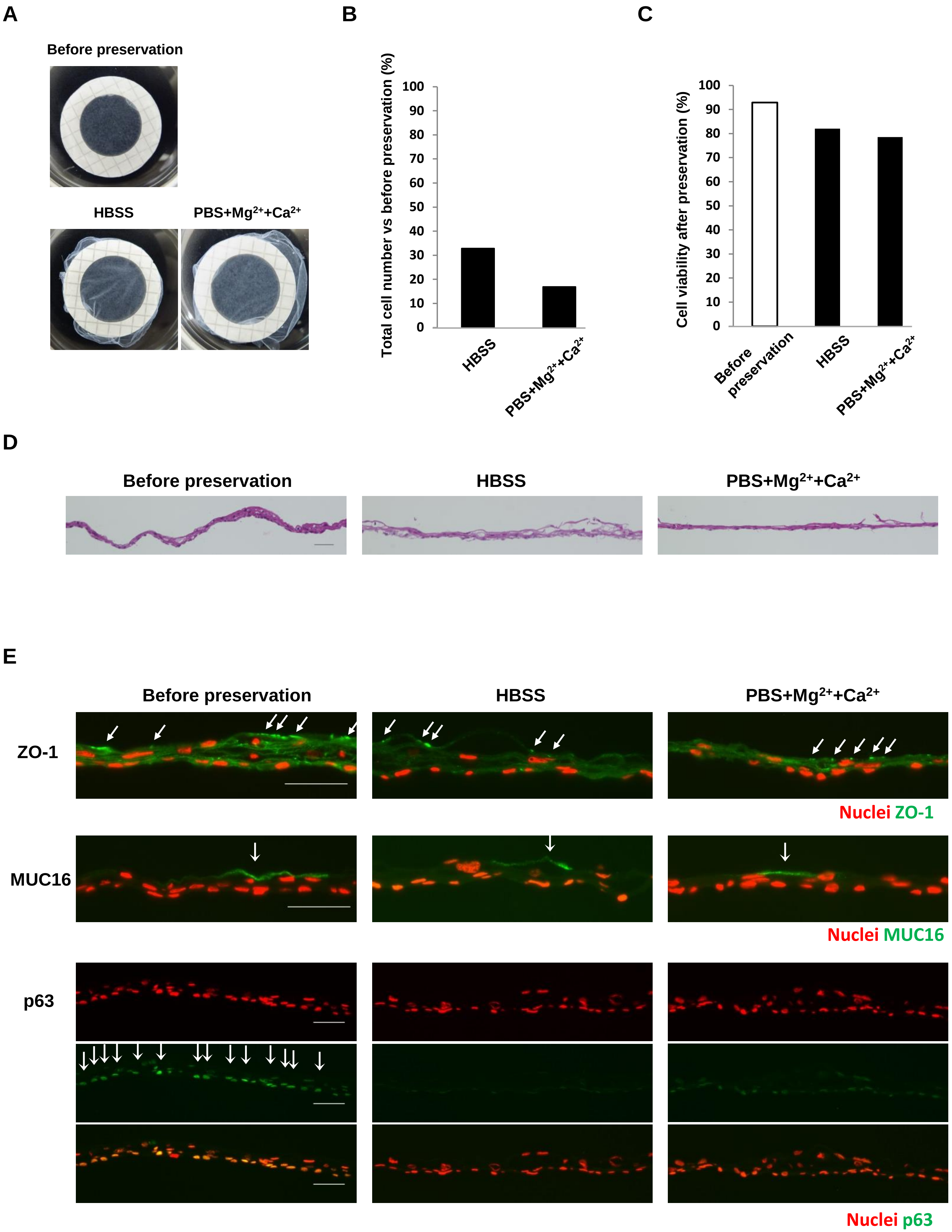

Supplement: Supplementary Information [file srep38987-s1.pdf]
